# Whole-genome characterization of clinical *Stenotrophomonas maltophilia* in Lebanon: sequence types, resistome, and T6SS diversity

**DOI:** 10.1128/spectrum.01344-26

**Published:** 2026-09-18

**Authors:** Carmen Adam, Rayane Adam, Peter Saba, Rita Menhem, Nareg Assadourian, Thomas Gebrayel, Charbel Al Khoury, George F. Araj, Sima Tokajian

**Affiliations:** 1 Department of Biological Sciences, School of Arts and Sciences, Lebanese American University 114792, Byblos, Lebanon; 2 Department of Computer Science and Mathematics, School of Arts and Sciences, Lebanese American University 114792, Byblos, Lebanon; 3 Department of Biological Sciences, School of Arts and Sciences, Lebanese American University 851058, Beirut, Lebanon; 4 Department of Pathology and Laboratory Medicine, American University of Beirut, Medical Center 535385, Beirut, Lebanon; Central Texas Veterans Health Care System, Temple, Texas, USA

**Keywords:** *Stenotrophomonas maltophilia*, antimicrobial resistance, colistin resistance, virulence factors, type VI secretion system, mobile genetic elements

## Abstract

*Stenotrophomonas maltophilia* is an emerging multidrug-resistant opportunistic pathogen associated with high morbidity and mortality in hospitalized patients. We present an integrated genomic and phenotypic characterization of 57 clinical *S. maltophilia* isolates from a tertiary care hospital in Lebanon. Whole-genome sequencing defined resistance profiles, virulence traits, and mobile genetic elements. Multilocus sequence typing revealed marked diversity across 12 known sequence types, with more than half (56%) of isolates assigned to novel or untypable STs. Five ST138 isolates were recovered from respiratory samples in critical care units, a pattern consistent with nosocomial transmission. Phenotypically, 28.1% and 3.5% of isolates were resistant to levofloxacin and trimethoprim–sulfamethoxazole, respectively; 71.9% were non-susceptible to colistin; and one (1.8%) was non-susceptible to minocycline. Trimethoprim–sulfamethoxazole resistance was not associated with *sul1* or *sul2*, and fluoroquinolone resistance was only partially explained by *smeD*, pointing to multifactorial mechanisms. Virulence genes *fliC*, *smf-1*, and *katA* were broadly conserved, whereas biofilm formation varied markedly. Type I secretion system components *hlyB* and *hlyD* occurred in a minority of isolates, with *hlyD* detected on both chromosomal and plasmid loci in three. A complete type VI secretion system (T6SS) core was identified in four isolates, with VipA subtyping placing most in i4b and a divergent CASM6 variant in i3. Tn*3* family transposons linked to metal resistance genes were carried by 17 isolates. Together, these findings reveal a genetically diverse population in which multifactorial resistance, heterogeneous T6SS carriage, variable biofilm capacity, and transposon-linked metal resistance converge to shape clinical adaptability, emphasizing the need for integrated surveillance to guide treatment and infection control.

**IMPORTANCE:**

*Stenotrophomonas maltophilia* has emerged as an important cause of infections globally. This is largely due to its intrinsic resistance and genomic plasticity. Through integrated genomic and phenotypic characterization of 57 clinical isolates, this study reveals a highly diverse population with a stable core genome and a dynamic accessory genome shaped by mobile genetic elements. The combination of widespread intrinsic resistance, variable acquired resistance traits, and virulence factors highlights the adaptive pathogenic capacity of this organism. These results highlight the importance of combining genomic data with phenotypic analysis to better understand pathogen evolution and transmission. By providing the first comprehensive molecular overview of clinical *S. maltophilia* isolates from Lebanon, this study supports the implementation of genomic surveillance frameworks and informed infection control strategies to limit the spread of this multidrug-resistant pathogen.

## INTRODUCTION

*Stenotrophomonas maltophilia* is a gram-negative bacterium that has gained increasing clinical attention for its high-pathogenicity role in nosocomial infections globally, including Lebanon (1, 2). The genus name reflects the organism’s specialized ability to grow on a limited range of substrates, highlighting its adaptability across both clinical and environmental niches (3). Beyond its clinical relevance, *S. maltophilia* is also found in diverse environmental niches, where it contributes to bioremediation processes and promotes plant growth (4), revealing its ecological versatility rather than its mechanisms of infection or transmission. Clinically, *S. maltophilia* represents a serious concern because of its multidrug resistance (MDR) and its association with high mortality, reaching up to 69% in bacteremia cases (5). In an international prioritization exercise focused on intensive care units (ICU), *S. maltophilia* ranked among the 10 most important antimicrobial-resistant organisms and was classified within the medium-priority tier (6). This resistance pattern is linked to a combination of intrinsic and acquired mechanisms (5, 7).

*S. maltophilia* shows increasing resistance to multiple antibiotic classes. Acquired resistance to polymyxins, including colistin and polymyxin B, has been reported, with colistin resistance estimated at 42% among clinical isolates (8, 9). Although trimethoprim–sulfamethoxazole (SXT) remains the first-line therapy (10), resistance to SXT is also emerging, largely driven by plasmid-mediated horizontal gene transfer involving insertion sequence common region elements and the *sul1* and *sul2* genes (11, 12). In addition to SXT, levofloxacin (LVX) and minocycline (MNO) are currently among the main alternative antibiotics used for the treatment of *S. maltophilia* infections (13). In parallel, *S. maltophilia* displays intrinsic resistance to β-lactams through the inducible β-lactamases L1 and L2, which together confer resistance to carbapenems, extended-spectrum cephalosporins, and aztreonam (14–16).

Beyond resistance, *S. maltophilia* carries a diverse set of virulence factors that support colonization and trigger host immune responses, including biofilm formation, adhesion, quorum sensing, and swarming (16–18). However, despite its recognition as an MDR pathogen with limited treatment options, it remains underexplored, particularly regarding the interplay between resistance mechanisms, virulence factors, and genetic diversity that drives its adaptability across clinical and environmental settings (19). These features, along with its association with high mortality and its ability to modulate co-infecting microorganisms, emphasize the need for in-depth molecular investigation. Given the limited molecular data on this pathogen in Lebanon, we performed a molecular characterization of 57 clinical *S. maltophilia* isolates, focusing on antimicrobial resistance profiles, virulence determinants, the type VI secretion system (T6SS), and mobile genetic elements, including insertion sequences and transposons. This work adds to the global understanding of this pathogen and provides context-specific insights into its molecular and epidemiological features in Lebanon.

## MATERIALS AND METHODS

### Bacterial isolates

A total of 57 non-duplicate *S. maltophilia* isolates were recovered from clinical specimens submitted for culture at the Clinical Microbiology Laboratory (CML), Department of Pathology and Laboratory Medicine, American University of Beirut Medical Center, between 2018 and 2021. This CML has been accredited by the College of American Pathologists since 2004. The isolates were identified using matrix-assisted laser desorption ionization–time of flight mass spectrometry using the MALDI Biotyper software (Bruker Daltonics, Bremen, Germany). Each isolate was designated with the prefix CASM followed by a unique identification number. The isolates were obtained from different clinical sources, including deep tracheal aspirate, blood, urine, tissue, abdominal, sputum, bone, wound, abscess, and bronchial specimens. The patients’ ages ranged from 27 days to 91 years, with a mean age of 60 years (Table S1). The cohort comprised 22 female and 35 male patients. All bacterial isolates were cryopreserved in a glycerol-based medium and stored at −80°C.

### Antimicrobial susceptibility testing

The antimicrobial susceptibility of each *S. maltophilia* isolate was tested using the Kirby–Bauer disk diffusion method and interpreted following the Clinical and Laboratory Standards Institute (CLSI) guidelines (20). Three antimicrobial agents (Bio-Rad, Hercules, CA, USA) were tested against each isolate: LVX, SXT, and MNO (20).

The minimum inhibitory concentration (MIC) for colistin was determined using the broth microdilution method, following the CLSI guidelines (20). *Escherichia coli* ATCC 25922, a colistin-susceptible strain, was used as a negative control. According to CLSI guidelines for *Pseudomonas aeruginosa*, isolates with MICs ≥4 µg/mL should be considered colistin resistant (13).

### Biofilm formation assay

A fresh bacterial liquid culture was incubated at 37°C for 24 h in Luria broth supplemented with 2% filtered glucose. Cultures were diluted 1:100 and inoculated into flat-bottom 96-well microtiter plates, followed by 24 h incubation at 37°C. After 24 h, wells were emptied and washed twice to remove non-adherent cells. Biofilms were stained with 0.1% crystal violet, rinsed repeatedly to remove excess stain, and allowed to air-dry for 5–24 h. Subsequently, 125 µL of 30% acetic acid was added to each well to solubilize the bound dye, and absorbance was measured at 595 nm using a Multiskan FC Microplate Photometer (Thermo Fisher Scientific, USA) (21). Optical density values were analyzed using SkanIt software, and biofilm classification was performed (22) based on the calculated optical density cut-off value (ODc), determined as follows:

ODc = OD_average of negative control_ + (3 × standard deviations).

Biofilm production was classified according to the following criteria:

non-producer: OD ≤ ODc, weak producer: ODc < OD ≤ 2 × ODc, moderate producer: 2 × ODc < OD ≤ 4 × ODc, strong producer: OD > 4 × ODc.

### Short- and long-read whole-genome sequencing

DNA extraction was performed using the Sigma Aldrich extraction kit following the manufacturer’s instructions for all the isolates. The extracted DNA was stored at −20°C. Genomic libraries were prepared using the Illumina Nextera XT DNA Library Preparation Kit with Dual Indexing (Illumina). The genomic DNA underwent end repair and A tailing, followed by ligation with adapters and sample-specific barcodes. Libraries were then multiplexed, clustered, and sequenced on the Illumina MiSeq platform (Illumina, San Diego, CA, USA) using a paired-end protocol with a read length of 2 × 300 base pairs (23).

High-molecular-weight (HMW) DNA was extracted using the MagAttract HMW DNA Kit (QIAGEN), following the manufacturer’s instructions. Nanopore sequencing was performed using the Ligation Sequencing Kit (SQK-LSK109; Oxford Nanopore Technologies [ONT], Oxford, UK) in combination with the Native Barcoding Kit (EXP-NBD104). Amplicon preparation included DNA end repair and barcode ligation, using 100–200 fmol of undiluted sample. Adapter ligation and purification were carried out using NEB ligation reagents and AMPure XP beads (Beckman Coulter, USA). After ligation, 50–100 fmol of the final DNA library was pooled and loaded onto R10.4.1 flow cell (ONT) for sequencing on the MinION platform (Oxford Nanopore Technologies). The raw Oxford Nanopore sequencing data were basecalled with Dorado v0.7.0 (ONT) using the fast basecalling model dna_r10.4.1_e8.2_400bps_fast@v4.2.0 (https://github.com/nanoporetech/dorado?tab=readme-ov-file#dna-models).

### Data analysis

#### Sequencing data processing and genome assembly workflow

The quality of the raw Illumina short-read sequences for all 57 isolates was assessed using FastQC v0.11.9 (24), and adapter trimming was performed with Trimmomatic v0.39 (25). *De novo* genome assembly of Illumina reads was conducted using SPAdes v3.15.5 (26). Five isolates (CASM7, CASM26, CASM33, CASM106, and CASM81) were selected as representatives based on their molecular profiles and were additionally sequenced on the Oxford Nanopore platform; the remaining 52 isolates had short-read data only. Long-read assemblies were generated from the basecalled Nanopore reads using Flye (27). Assembly quality and genome completeness were assessed with QUAST v5.2.0 (28) and BUSCO v6.1.0 (29), using the xanthomonadales_odb12 lineage data set. Mean coverage of the short and long reads was taken from the SPAdes and Flye output files, respectively. For the five isolates with both data types, Illumina and Nanopore reads were combined into hybrid assemblies using Unicycler v0.5.0 (30), which were evaluated with QUAST and BUSCO as described above.

Genome annotation was performed using the Prokaryotic Genome Annotation Tool, Prokka (31). *In silico* analyses were carried out using bioinformatics tools available through the Center for Genomic Epidemiology (CGE) (https://www.genomicepidemiology.org/). Species identification was performed using KmerFinder v3.1 (32), and sequence types were assigned using the CGE multilocus sequence typing tool (MLST) (https://cge.food.dtu.dk/services/MLST/). Antimicrobial resistance genes were identified using ResFinder v4.1 (https://genepi.food.dtu.dk/resfinder) and the Comprehensive Antibiotic Resistance Database (33). Virulence-associated genes were annotated based on Prokka outputs. Mobile genetic elements, including insertion sequences, were detected using MobileElementFinder v1.0.3 (https://cge.food.dtu.dk/services/MobileElementFinder/). Visualization of metal resistance genes and insertion sequence contexts was performed using SnapGene Viewer (snapgene.com). For synteny analysis, CASM6, CASM34, and CASM48 were selected to represent the Tn*3* genetic environment and were compared using Clinker v0.0.32 (34). The visualized region spanned a 60 kbp window ending at the shared IS*Pa34* element and included only the functionally annotated genes identified by Prokka.

#### Core genome phylogenetic analysis

A core genome-based maximum-likelihood phylogenetic tree was constructed using 57 *S. maltophilia* clinical isolates together with 10 publicly available reference genomes retrieved from the National Center for Biotechnology Information. The reference genomes included SM_CYZ (NZ_CP101622.1), SM_SG.Y2 (NZ_CP106759), SM_SKK55 (NZ_CP040433.1), SM_ICU331 (NZ_CP040440), SM_D457 (HE798556), SM_NCTC10258 (NZ_LS483377.1), SM_JV3 (NC_015947), SM_K279a (NC_010943.1), SM_ORO2 (NZ_CP056088.1), and SM_R551-3 (CP001111.1). Genome annotation was performed using Prokka (31). Core genome alignment was generated with Roary (35). The Roary core genome comprised genes present in ≥99% of the genomes, corresponding to the intersection of the Prokka-annotated genes across the 57 study isolates and the 10 reference genomes. Orthologous genes were identified by pairwise BLASTP comparison using a 95% identity threshold.

The maximum-likelihood tree was inferred using FastTree v2.1.11 (36). The resulting phylogeny was visualized using iTOL v5 (37).

### T6SS gene profiling and comparative analysis

T6SS gene content was characterized in the 57 *S. maltophilia* isolates, together with two previously described T6SS-positive reference strains, SKA14 (NZ_ACDV00000000) and SKK55 (NZ_CP040433.1). Genome annotation was performed using Prokka (31), followed by identification of T6SS core components using HMMER v3 (38) with a pre-built hidden Markov model specific for T6SS. The targeted genes included *tssA* (COG3515), *tssB* (*vipA*, COG3516), *tssC* (*vipB*, COG3517), *hcp* (*tssD*, COG3157), *vgrG* (*tssI*), *clpV* (*tssH*), and *dotU* (*tssL*, COG3455). Identified sequences were aligned using MAFFT v7.471 (39) to ensure accurate multiple sequence alignment. Evolutionary relationships among T6SS gene clusters were inferred using IQ-TREE under a maximum-likelihood framework (40), and trees were visualized with iTOL v5 (37). T6SS subtypes were assigned using SecReT6 v3 (41).

## RESULTS

### *In silico* species identification and sequence type distribution

**Fig 1:**
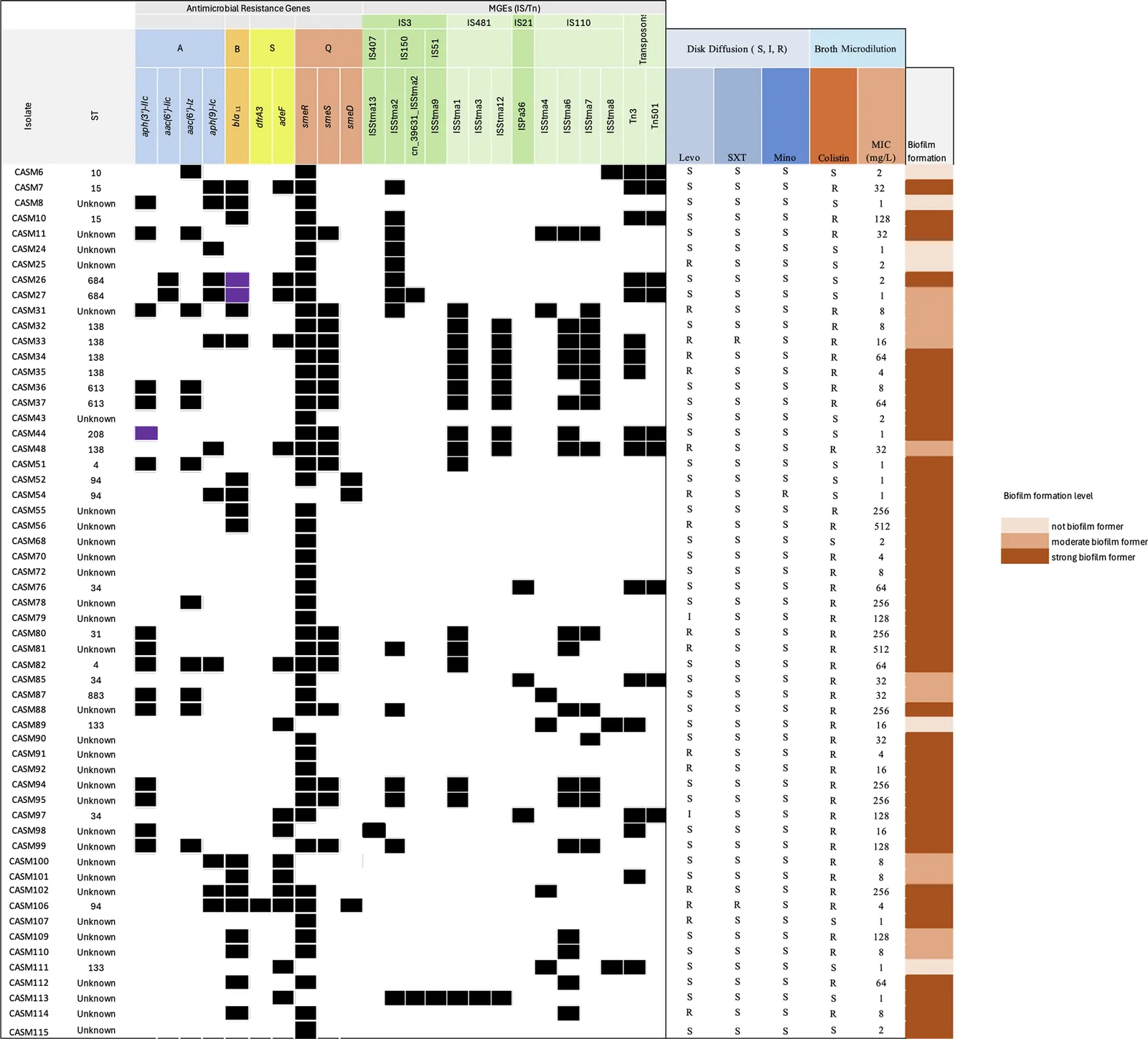
Antimicrobial resistance, molecular characteristics, and biofilm formation among the 57 *S. maltophilia* isolates. Antimicrobial resistance was assessed using *in silico* detection of resistance genes and antimicrobial susceptibility testing by disk diffusion and Broth Microdilution for colistin. Sequence types, insertion sequences, and transposons were identified *in silico*. Biofilm formation was classified as non-producer, moderate producer, or strong producer. Resistance genes are grouped as follows: A, aminoglycoside resistance genes; B, β-lactam resistance genes; S, trimethoprim–sulfamethoxazole resistance genes; and Q, fluoroquinolone resistance genes. Genes associated with the Tn*501* transposon are highlighted with a purple box. Black boxes indicate the presence of a gene or mobile genetic element, whereas white boxes indicate its absence. Antimicrobial susceptibility results are reported as susceptible (S), intermediate (I), or resistant (R) according to CLSI guidelines. Colistin minimum inhibitory concentration (MIC) values were interpreted using *Pseudomonas aeruginosa* breakpoints.

Species identification of all 57 *S. maltophilia* isolates was first confirmed using KmerFinder following WGS. Subsequently, *in silico* MLST was performed based on the sequences of seven housekeeping genes: *atpD*, *gapA*, *guaA*, *mutM*, *nuoD*, *ppsA*, and *recA*. Twent-five isolates were assigned to 12 different known STs; the remaining 32 (56%) could not be assigned to a recognized ST (Fig. 1). The remaining isolates were distributed as follows: ST10 (*n* = 1), ST133 (*n* = 2), ST15 (*n* = 2), ST94 (*n* = 3), ST684 (*n* = 2), ST138 (*n* = 5), ST613 (*n* = 2), ST208 (*n* = 1), ST4 ( *n* = 2), ST31 (*n* = 1), ST883 (*n* = 1), and ST34 (*n* = 3).

### Resistance determinants and phenotypic susceptibility patterns

A diverse repertoire of antimicrobial resistance determinants among the 57 *S. maltophilia* isolates was detected (Fig. 1), with substantial heterogeneity observed both within and across sequence types. Aminoglycoside resistance genes were variably distributed, with *aph(3*′*)-IIc* detected most frequently (28.1%), followed by *aph(9)-Ic* (21.1%), and *aac(6*′*)-Iz* (19.3%) and the β-lactamase gene *bla*_L1_ was present in 33.3% of isolates and occurred across multiple sequence types, including ST138, ST684, and unassigned types. Trimethoprim resistance genes were rare, with *dfrA3* detected in a single isolate, whereas *tolC* was detected in most (98.2%). Fluoroquinolone-associated efflux regulators were widespread, with *smeR* found in 87.7% of isolates, whereas *smeS* and *smeD* occurred less frequently. Core MDR and regulatory genes, including *macA*, *macB*, *emrA*, *emrB*, and *phoP*, were present in all isolates.

*bla*_L1_ was associated with Tn*501* transposon in two ST684 isolates and *aph(3*′*)-IIc* carried on the same transposon in an ST208 isolate, supporting localized mobilization events rather than lineage-specific inheritance.

Phenotypic testing showed resistance to levofloxacin in 28.1% of isolates and resistance to trimethoprim–sulfamethoxazole in two isolates. Minocycline resistance was limited to a single isolate. Colistin resistance was highly prevalent, with 71.9% of isolates exhibiting MICs of ≥4 µg/mL. Non-susceptible isolates were distributed across various sequence types.

### Distribution and genomic context of mobile genetic elements

Mobile genetic elements were common among the 57 *S. maltophilia* isolates, with 68.4% (*n* = 39) carrying at least one IS and 29.8% (*n* = 17) harboring transposons (Fig. 1). The most prevalent IS was IS*Stma6*, detected in 31.6% (*n* = 18) of isolates, followed by IS*Stma1* in 28.1% (*n* = 16), IS*Stma7* in 26.3% (*n* = 15) and IS*Stma2* in 24.6% (*n* = 14). Several ISs were isolate specific, including IS*Stma13* in CASM98 and IS*Stma9* and IS*Stma3* in CASM113. In contrast, ISs were not detected in several isolates (CASM8, CASM43, CASM52, CASM54, CASM55, CASM56, CASM68, CASM70, CASM72, CASM78, CASM79, CASM91, CASM92, CASM100, CASM101, CASM106, CASM107, and CASM115).

**Fig 2:**
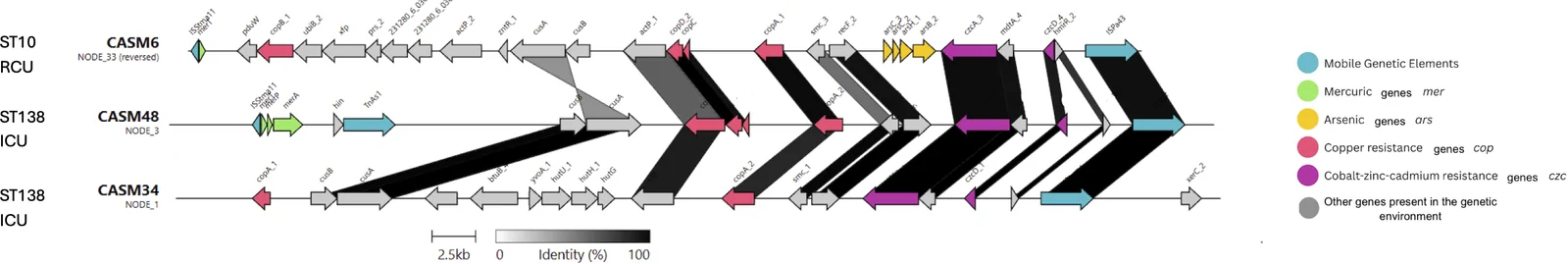
Genetic environment of IS*Pa43* (Tn*3* family transposase) in isolates CASM6, CASM34, and CASM48. Arrows represent annotated open reading frames, and genes are color-coded according to functional category. In CASM6, IS*Pa43* is co-localized with multiple metal resistance determinants, including copper resistance genes (*copA*, *copB*, *copC*, and *copD*), arsenic resistance genes (*arsB*, *arsC*, and *arsH*), cobalt–zinc–cadmium resistance genes (*czcA* and *czcD*), and a mercuric resistance gene (*merT*). CASM48 harbors a highly dynamic region comprising Tn*As1*, IS*Pa43*, and IS*Stma11*, associated with cobalt–zinc–cadmium resistance genes (*czcA* and *czcD*), copper resistance genes (*copA*, *copC*, and *copD*), and mercuric resistance genes (*merA*, *merP*, and *merT*). In contrast, CASM34 lacks the insertion sequence IS*Stma11* and mercuric resistance genes and contains only cobalt–zinc–cadmium resistance genes (*czcA* and *czcD*) and copper resistance genes (*copA*, *copC*, and *copD*). The sequence types (STs) and clinical source (RCU or ICU) are shown for each isolate. The genomic region visualized is defined within a 60 kbp window ending with the common IS*Pa34* element, encompassing only the functionally identified genes that are annotated by Prokka. Clinker was used for synteny analysis. The scale bar (2.5 kb) is included for reference and identity threshold in percent.

Analysis of the surrounding genetic contexts revealed that 17 isolates carried elements from the Tn*3* transposon family, frequently embedded within regions enriched in metal resistance genes. These regions commonly included mercury resistance genes (*merA*, *merP*, and *merT*), copper resistance genes (*copA*, *copC*, and *copD*), arsenic resistance genes (*arsB* and *arsC*), and cobalt–zinc–cadmium resistance genes (*czcA* and *czcD*), together with hypothetical proteins. Structural comparison across isolates revealed substantial variability in gene content and organization. In CASM6, a Tn*3* family transposase (IS*Pa43*) co-localized with multiple metal resistance determinants, whereas CASM48 harbored a highly dynamic region containing Tn*As1*, IS*Pa43*, and IS*Stma11* in combination with several metal resistance genes. CASM34, however, lacked IS*Stma11* and the mercury resistance genes. Collectively, these patterns indicate substantial genomic plasticity, with mobile genetic elements contributing to the modular assembly and diversification of resistance-associated regions in this population (Fig. 2).

### Distribution of virulence-associated genes across *S. maltophilia* isolates

**Fig 3:**
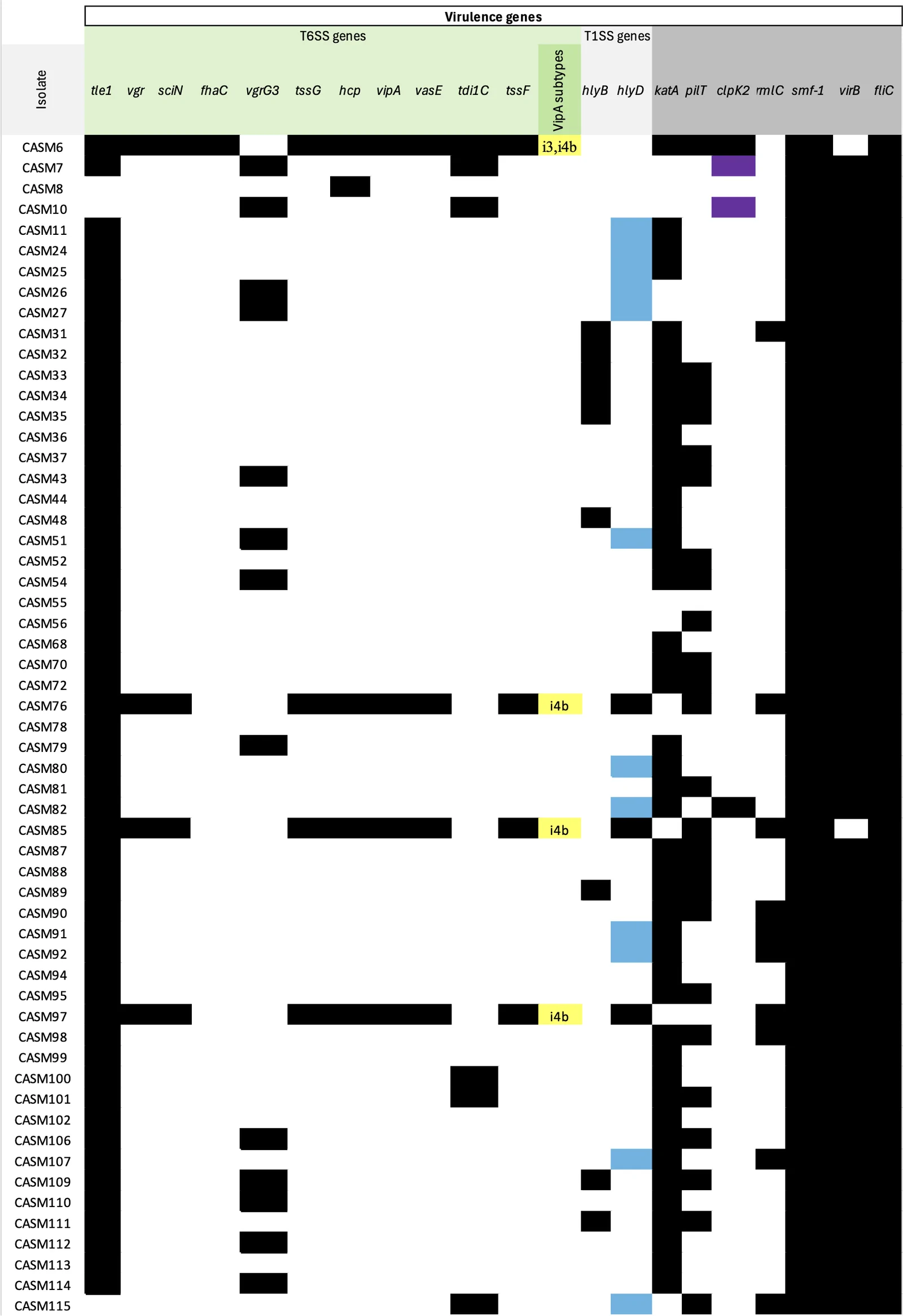
*In silico* detection of virulence genes, including key T1SS and T6SS genes. The presence of *hlyB*, *hlyD*, and T6SS core genes is shown, with T6SS types classified based on VipA subtyping as i4b, with subtype i3 found only in CASM6. Blue indicates *hlyD* detected on both chromosomal and plasmid locations, whereas purple indicates *clpK2* associated with the Tn*501* transposon. The black box indicates the presence of the gene, and the white box indicates the absence.

*In silico* analysis of virulence-associated genes across the 57 *S. maltophilia* isolates revealed a conserved core of virulence traits alongside variable accessory factors (Fig. 3). The motility and adhesion genes *fliC* and *smf-1* were detected in all isolates, indicating a conserved capacity for flagellar motility and surface attachment. The type IV secretion system component *virB* was present in 96.5% (*n* = 55) of isolates, making it a near-universal feature of the collection.

Genes associated with stress tolerance and motility showed greater heterogeneity. The catalase gene *katA* was identified in 78.9% (*n* = 45) of isolates, whereas *pilT* was detected in 43.9% (*n* = 25). The *rmlC* gene, linked to L-rhamnose biosynthesis and biofilm formation, was present in 19.3% (*n* = 11). The heat shock–associated gene *clpK2* was rare, occurring in four isolates, with co-localization with Tn*501* observed in two cases, supporting a role for mobile genetic elements in its acquisition.

*hlyD* and *hlyB*, which encode components of the type I secretion system (T1SS) associated with α-hemolysin secretion in gram-negative bacteria, were detected in 21.1% (*n* = 12) and 15.8% (*n* = 9) of isolates, respectively, with three isolates carrying both chromosomal and plasmid-associated copies of *hlyD*. This gene combination suggests the potential for hemolysin-mediated cytotoxicity in a limited fraction of the collection.

### Core T6SS gene phylogeny and VipA subtype diversity

**Fig 4:**
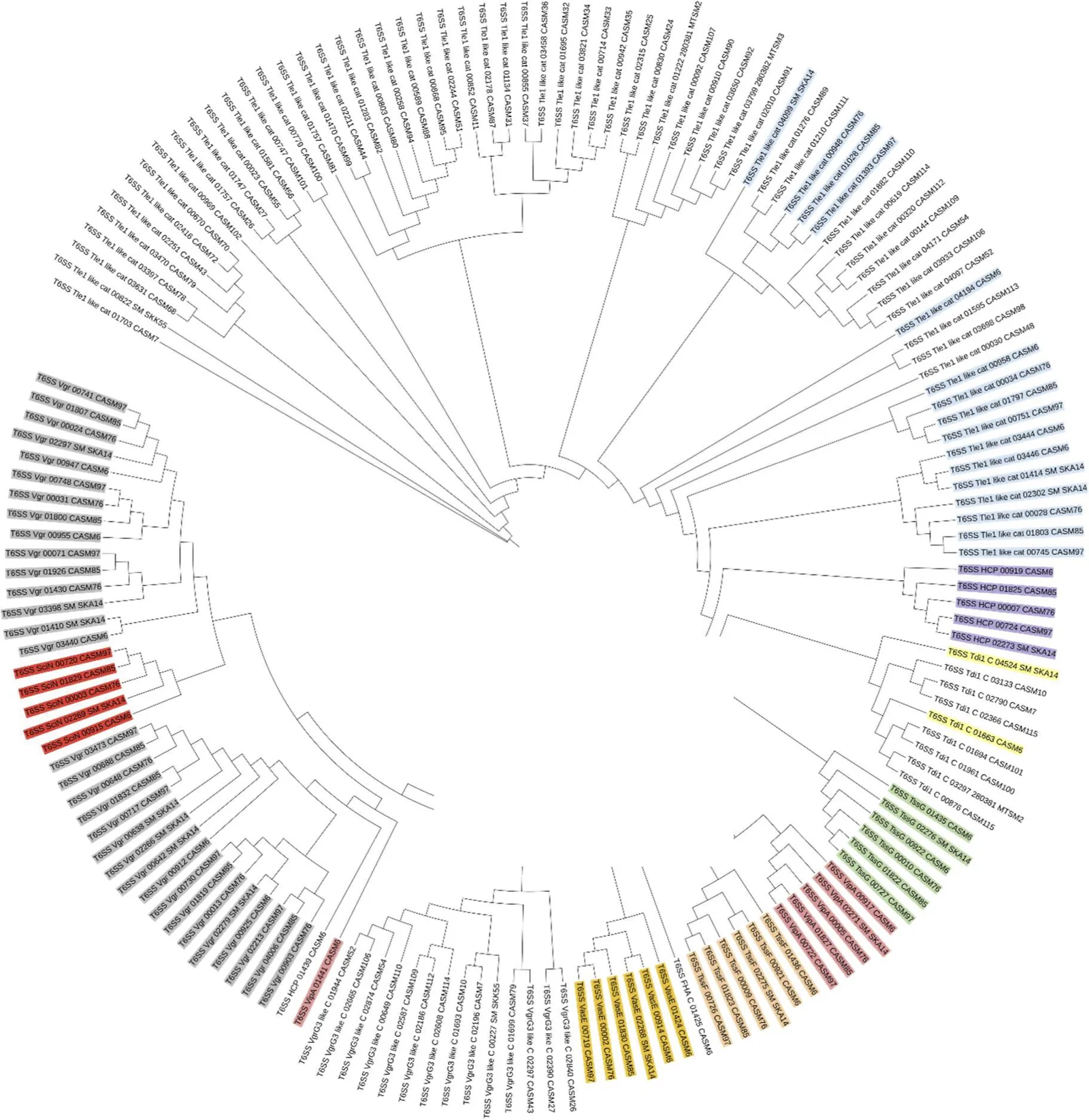
Phylogenetic distribution of type VI secretion system (T6SS)-related genes in *S. maltophilia* isolates. Color-coded representation of T6SS genes in VipA-positive isolates CASM6, CASM76, CASM85, CASM97, and SKA14: Tle1 (blue), Hcp (purple), Tdl1C (yellow), TssG (green), VipA (pink), TssF (light orange), VasE (orange), Vgr (gray), and SciN (red). The number between the protein name and the isolate name indicates the position of that amino acid sequence in the genome.

A phylogenetic tree based on core T6SS genes was constructed using homology-based identification and included two reference strains, SKA14 and SKK55 (Fig. 4). Five isolates, CASM6, CASM76, CASM85, CASM97, and SKA14, clustered together and carried a complete set of core T6SS components, including Tle1, Hcp, Tdl1C, TssG, VipA, TssF, VasE, Vgr, and SciN. Multiple variants of Tdl1C and Vgr were detected within these isolates, indicating diversity in T6SS effector and structural elements.

**Fig 5:**
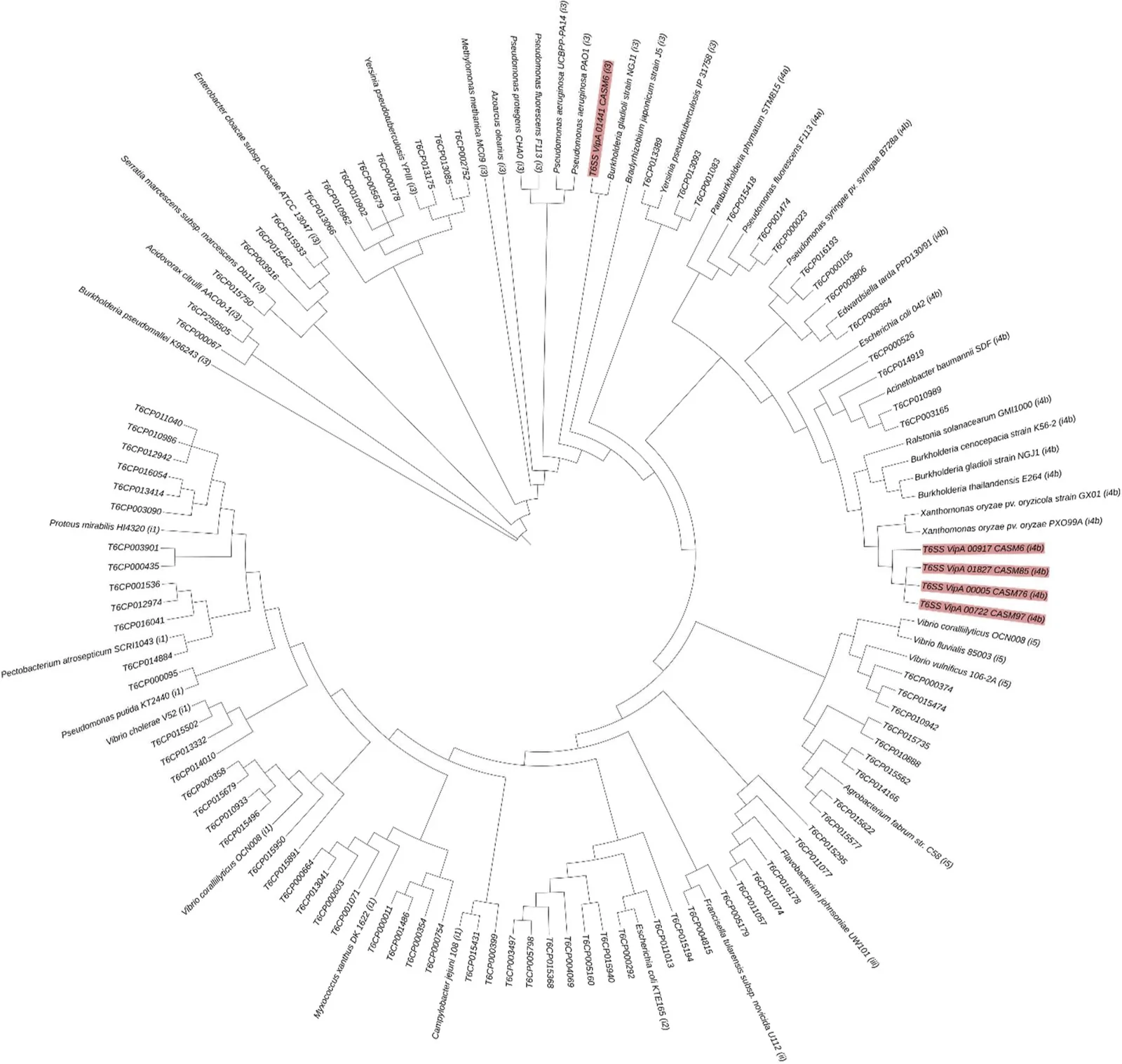
Phylogenetic tree showing the relationship between the query VipA protein and experimentally validated VipA proteins recorded in the SecReT6 database. VipA sequences identified in isolates from this study are highlighted with a pink background. The remaining sequences correspond to experimentally validated VipA proteins from diverse bacterial genera, including *Xanthomonas*, *Acinetobacter*, *Burkholderia*, *Pseudomonas*, *Escherichia*, *Vibrio*, *Myxococcus*, *Campylobacter*, *Pectobacterium*, *Proteus*, *Acidovorax*, *Serratia*, *Enterobacter*, *Yersinia*, *Methylomonas*, *Azoarcus*, *Flavobacterium*, *Francisella*, *Agrobacterium*, and *Edwardsiella*. T6SS subtype designations (i1, i2, i3, i4a, i4b, i5, ii, and iii) are indicated.

Subtype classification based on VipA sequences showed that only CASM6, CASM76, CASM85, and CASM97 encoded VipA, with CASM6 harboring two distinct variants. One VipA variant in CASM6 clustered with VipA proteins from *Burkholderia gladioli* NGJ1 and belonged to subtype i3. The remaining VipA sequences, from CASM6, CASM76, CASM85, CASM97, and SKA14, grouped with reference strains of *Xanthomonas*, *Burkholderia*, and *Ralstonia* and were classified as subtype i4b (Fig. 5). These i4b proteins were 166 amino acids long and shared 96%–97% sequence identity, whereas the second CASM6 variant showed only 39% identity to them, consistent with its assignment to subtype i3.

### Biofilm formation

**Fig 6:**
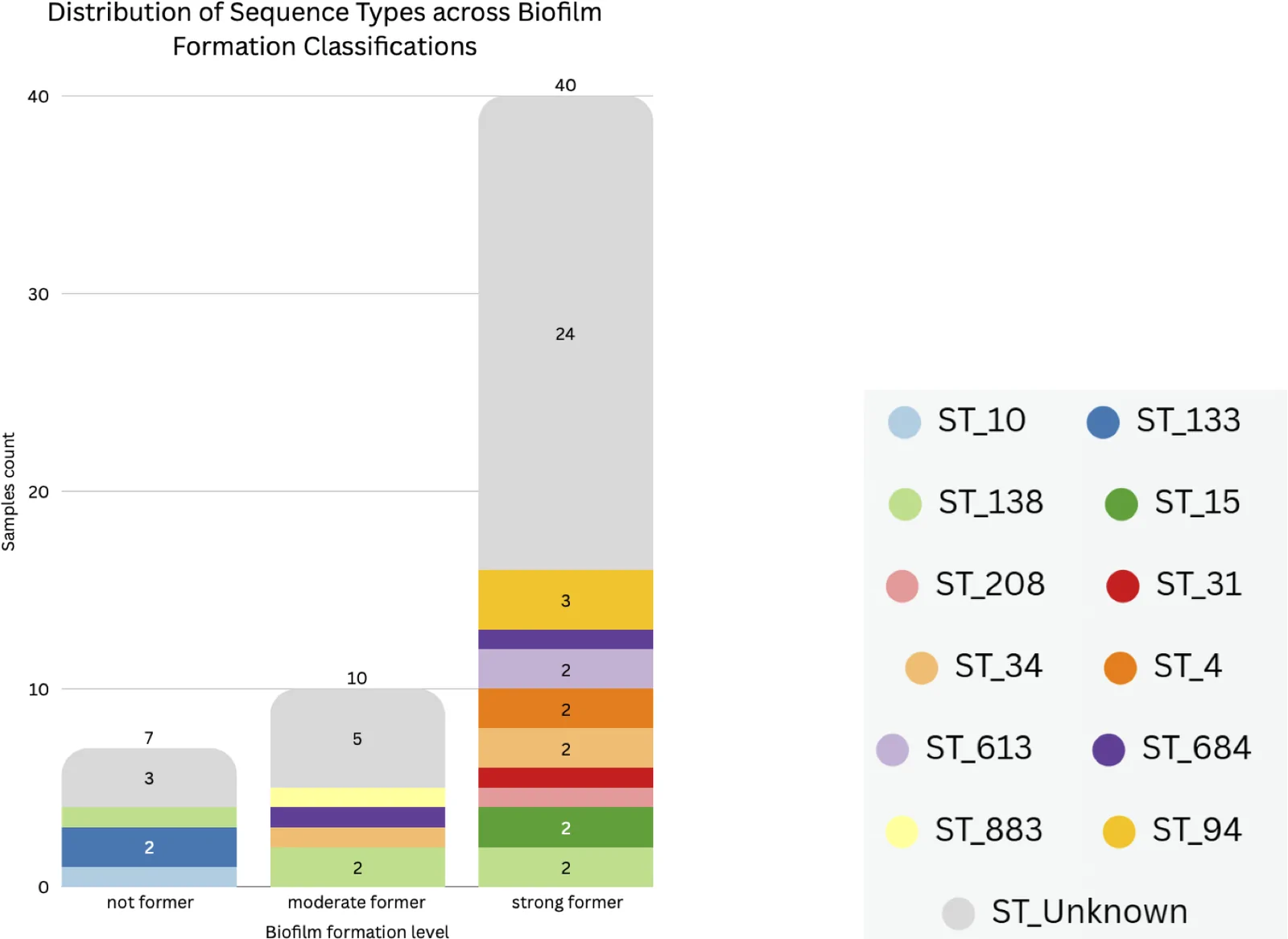
Distribution of sequence types across biofilm formation levels among *S. maltophilia* isolates. Stacked bars represent the number of isolates classified as non-producers, moderate producers, or strong producers, with individual colors indicating the corresponding sequence types (STs). The numbers within each colored segment indicate the number of isolates belonging to each ST within the respective biofilm formation category. Isolates for which a sequence type could not be assigned are shown in gray as ST_Unknown. The total number of isolates in each biofilm formation category is indicated above each bar.

Most isolates were capable of biofilm formation (*n* = 50). According to the previously proposed classification scheme (22), 70.2% (*n* = 40) were strong biofilm producers; 17.5% (*n* = 10) were moderate producers; and 12.3% (*n* = 7) were non-biofilm producers distributed across the different resistance patterns and sequence types (Fig. 1 and 6).

### Core genome phylogenetic analysis

**Fig 7:**
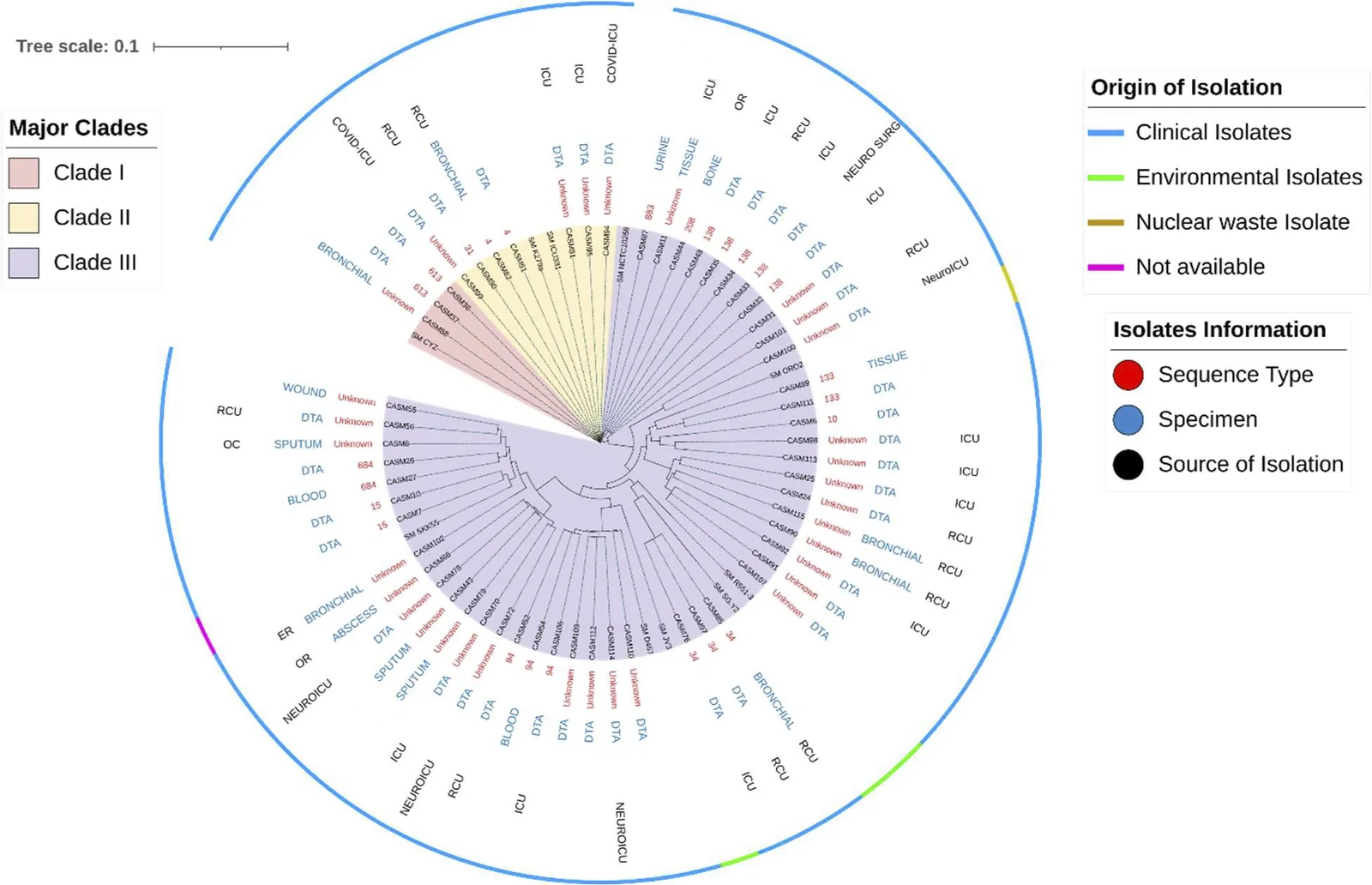
A maximum-likelihood phylogenetic tree was constructed based on the core genome alignment of the 57 clinical *S. maltophilia* isolates and 10 reference genomes from various origins. The tree resolves three major clades; each of the 57 isolates is annotated with its sequence type, specimen type, and hospital unit of isolation. The origin of isolation, clinical, environmental, or nuclear waste, is indicated for both the study isolates and the reference genomes. The core genome alignment was generated with Roary; the tree was inferred with FastTree; and the figure was visualized in iTOL. ER, emergency room; ICU, intensive care unit; NEUROSURG, neurosurgical ward; RCU, respiratory care unit.

A maximum-likelihood phylogenetic tree based on core genome alignments was constructed for the 57 *S. maltophilia* isolates together with 10 reference genomes from clinical, environmental, and nuclear waste sources (Fig. 7). The isolates clustered into three major clades. Each isolate was annotated with its sequence type, specimen type, and origin of isolation (Fig. 7). Clade III contained the largest number of isolates and included reference genomes from diverse origins, indicating substantial genomic diversity. Clade I comprised four isolates, including the clinical reference strain SM CYZ, whereas clade II included several well-characterized clinical reference strains, such as SM K279a, SM ICU331, and SM NCTC10258. The 57 Lebanese isolates distributed across all three clades and formed multiple subclades rather than a single country-specific lineage, indicating that clinical *S. maltophilia* from a single tertiary center spans a broad evolutionary landscape. Intermixing with clinical reference strains (SM K279a, SM ICU331, SM NCTC10258, and SM CYZ) and environmental references within the same subclades argues against a strict clinical-versus-environmental phylogenetic boundary and is consistent with cross-niche transmission or shared ancestry. The nuclear waste-associated SM ORO2 branched separately as a distinct lineage, an outlier shaped by niche-specific selection and indicating that extreme environments, rather than clinical settings, drive the most pronounced phylogenetic divergence in this species.

## DISCUSSION

This study integrates whole-genome sequencing with phenotypic characterization of 57 clinical *S. maltophilia* isolates from a single tertiary care hospital in Lebanon, linking sequence type diversity, antimicrobial resistance, virulence determinants, the T6SS, and mobile genetic elements to the adaptive capacity that underlies the organism’s success as an opportunistic pathogen. Two observations dominate the population structure. First, five ST138 isolates were recovered from deep tracheal aspirates of patients in the ICU, respiratory care unit, and neurosurgical wards, pointing to possible healthcare-associated transmission within critical care settings. Second, against this localized cluster, the collection was otherwise highly heterogeneous, with 32 isolates unassignable to previously defined sequence types, indicating that the population structure is shaped primarily by genetic diversification rather than by clonal dissemination. This dual signal of focal transmission on a background of pronounced genomic diversification provides the interpretive framework for the resistance, virulence, and mobile-element patterns examined below, each of which reflects both lineage-specific determinants and species-wide adaptive traits. Similar clustering of ST138 has been reported previously in ICU settings and linked to environmental reservoirs, such as contaminated sinks (42). Beyond this localized grouping, isolates in the present study were distributed across 12 known STs, with more than half remaining unassigned, reflecting extensive genetic diversity. This observation is consistent with large-scale studies reporting a high proportion of novel sequence types among clinical *S. maltophilia* isolates (43, 44) and reveals the limited resolution of sequence typing alone for epidemiological inference.

SXT, the recommended first-line therapy for *S. maltophilia* infections (10), retained activity across most of the collection, with resistance detected in only two isolates. Neither resistant isolate carried *sul1* or *sul2*, and only CASM106 harbored *dfrA3*, which offers a plausible genetic basis for its resistance; no known resistance determinant was identified in CASM33. Comparable findings have been reported in SXT-resistant *S. maltophilia* isolates lacking *sul-* or *dfr-*type genes (45), implicating alternative mechanisms. Among these, efflux-mediated resistance is most frequently implicated, with overexpression of the SmeDEF efflux pump reducing SXT susceptibility (46). Consistent with this, CASM106 also carried the *smeD* gene. However, the presence of *smeD* alone was not predictive of resistance, as additional *smeD*-positive isolates in our collection remained susceptible. This dissociation between gene carriage and phenotype suggests that gene carriage alone does not govern the SXT phenotype and is consistent with contributions from efflux pump expression, regulatory variation, or additional resistance determinants that were not assessed here, with contributions from outer membrane components such as TolCSm (47). Levofloxacin resistance was detected in 28.1% of isolates, a higher proportion than reported in several regional studies (48), and biofilm-associated tolerance may contribute to this elevated rate, given that biofilm growth confers fluoroquinolone tolerance in *S. maltophilia* (48). Efflux activity offers a complementary explanation: overexpression of *smeD* has been linked to fluoroquinolone resistance (49), and two of the three *smeD*-positive isolates in our collection were levofloxacin resistant. However, most levofloxacin-resistant isolates lacked *smeD*, pointing to a multifactorial basis for the phenotype. Additional mechanisms reported in *S. maltophilia*, including a chromosomally encoded *qnr* determinant capable of conferring low-level quinolone resistance (50), may also contribute and highlight the role of less well-characterized determinants and their regulation.

Minocycline retained strong *in vitro* activity against the collection, with 98.2% of isolates susceptible, in line with previous reports of comparable susceptibility rates among clinical isolates surveyed over an 8-year period (51). These findings reinforce its role as a valuable therapeutic alternative, particularly considering the adverse effects associated with trimethoprim–sulfamethoxazole and the reduced activity of fluoroquinolones in this cohort (52). Of relevance, CASM33, which was resistant to both trimethoprim–sulfamethoxazole and levofloxacin, remained susceptible to minocycline, illustrating its potential clinical utility in multidrug-resistant cases.

Colistin resistance was highly prevalent, with 71.9% of isolates exhibiting MICs ≥4 µg/mL, in line with increasing reports of diminished susceptibility in *S. maltophilia* (8, 9). Species-specific breakpoints are still lacking, and the relative contributions of intrinsic and acquired mechanisms remain debated (53); nevertheless, the genomic data are consistent with a predominantly intrinsic basis. All isolates carried *phoP*, which regulates lipid A modification, together with the *macAB* efflux system genes (54), indicating a conserved genetic background with the potential to contribute to polymyxin tolerance in this species.

Virulence gene profiling identified a conserved core of motility and adhesion determinants, with *fliC* and *smf-1* detected in all isolates. Detection of *smf-1* in every isolate is consistent with reports from multiple geographic regions describing widespread carriage of this fimbrial adhesin gene in clinical *S. maltophilia* (43, 55, 56). Despite this genetic uniformity, phenotypic assays revealed substantial variability in biofilm formation, ranging from strong producers to non-biofilm producers, indicating that *smf-1* presence alone is not sufficient to drive biofilm development. The low prevalence of *rmlC* (19.3%), which contributes to L-rhamnose biosynthesis and has been linked to biofilm formation, further supports the view that biofilm phenotypes in this collection arise from the combined action of multiple accessory genes and regulatory inputs rather than any single determinant (57).

The T1SS components *hlyB* and *hlyD* were detected in 15.8% and 21.1% of isolates, respectively. T1SS mediates the export of RTX-family proteins in gram-negative bacteria (58). Three isolates harbored *hlyD* on both chromosomal and plasmid loci, consistent with horizontal acquisition of an additional plasmid-borne copy. Although gene carriage does not establish hemolysin production, *hlyD* has been reported in *Stenotrophomonas* species as a candidate virulence determinant implicated in host–cell interactions (59), and its restricted distribution here is more consistent with strain-specific virulence strategies than with a conserved feature of *S. maltophilia* pathogenesis.

Profiling of the T6SS extended this virulence profile. A complete set of core T6SS components, including Tle1, Hcp, Tdl1C, TssG, VipA, TssF, VasE, Vgr, and SciN, was identified in five isolates (CASM6, CASM76, CASM85, CASM97, and the SKA14 reference), with multiple Tdl1C and Vgr variants suggesting effector and structural diversity within this subset. VipA subtyping placed CASM6, CASM76, CASM85, and CASM97 in subtype i4b together with *Xanthomonas*, *Burkholderia*, and *Ralstonia* references, whereas CASM6 uniquely carried a second, divergent VipA variant clustering with *Burkholderia gladioli* NGJ1 in subtype i3. This restricted distribution indicates that a fully assembled T6SS is an accessory rather than core feature of our collection. Its presence is nonetheless biologically significant. A recent survey of approximately 1,000 *S. maltophilia* genomes showed that T6SS loci occupy conserved genomic positions consistent with ancestral acquisition, and functional studies in a clinical sputum isolate confirmed Hcp secretion and T6SS-dependent killing of *Escherichia coli* and *Burkholderia cenocepacia*, together with competitive displacement of a co-infecting *Pseudomonas aeruginosa* strain, positioning the system as a contact-dependent antibacterial weapon that supports persistence in polymicrobial niches (60). In this context, the four T6SS-positive isolates in our cohort, particularly CASM6 with its two VipA variants, are strong candidates for enhanced interspecies competitiveness in the airway microenvironment, and the co-occurrence of a complete T6SS with a diverse accessory resistance and virulence repertoire points to an integrated adaptive arsenal that may contribute to the persistence of specific *S. maltophilia* lineages in critically ill patients.

Mobile genetic elements made a substantial contribution to the accessory genome. Tn*3* family transposons carrying metal resistance genes, including *merA*, were identified in 17 isolates; by contrast, plasmid-associated *merA*, previously reported in environmental isolates (61), was absent from this clinical collection, suggesting a chromosomal/transposon-mediated route of acquisition. The co-occurrence of IS*Stma11* with these clusters, as well as its absence from isolates with distinct profiles, is consistent with IS and transposon-mediated assembly of metal resistance regions and marks mobile genetic elements as a principal driver of accessory genome architecture in this collection.

The availability of 57 *S. maltophilia* genomes from different clinical sample types and hospital units, representing diverse antimicrobial resistance and virulence profiles, provides a valuable foundation for future pangenome analysis. Given the ecological versatility and adaptability of *S. maltophilia* to diverse environments, future comparison of the core and accessory genomes of these 57 isolates could provide insights into the genomic diversity and identify accessory genes potentially associated with antimicrobial resistance and virulence. Expanding such analyses to include environmental *S. maltophilia* isolates could further enable comparison of genomic features shared between clinical and environmental populations. Thus, this broader pangenome approach could be useful for future whole-genome sequencing studies and genomic surveillance and could facilitate investigations into the evolution and adaptation of *S. maltophilia*.

### Conclusion

By integrating whole-genome sequencing with systematic phenotypic characterization of 57 clinical *S. maltophilia* isolates, this study fills a critical surveillance gap for Lebanon and the wider Middle East and reframes how the clinical threat of this pathogen should be monitored. The population was dominated by novel sequence types on a stable core genome, with an accessory compartment actively reshaped by insertion sequences and Tn*3* family transposons, including horizontally acquired mercury resistance clusters and sporadic virulence determinants, such as *hlyD*. A fully assembled T6SS was present in five isolates and, given its established role as a contact-dependent antibacterial weapon supporting persistence in polymicrobial niches, likely contributes to the interspecies competitiveness of these strains in the airway environment. Phenotypic resistance to colistin, trimethoprim–sulfamethoxazole, and fluoroquinolones, much of it not fully explained by established resistance determinants, demonstrates that genotype-based prediction alone is insufficient to guide therapy for *S. maltophilia*. These results position *S. maltophilia* as a pathogen sustained by continuous genomic remodeling rather than clonal expansion, establish a regional genomic baseline, and argue for integrated genomic, functional, and epidemiological surveillance as the foundation for stewardship and infection control in at-risk patient populations.

## Data Availability

The 57 clinical isolates of *S. maltophilia* genomes generated and analyzed in this study have been deposited in the NCBI repository under BioProject accession number PRJNA1017353. Accession numbers and genome statistics of each isolate are listed and summarized in Table S2 for short reads and Table S3 for long reads and hybrid assemblies. The accession numbers of the additional reference genomes used for the phylogenetic trees are available within the Materials and Methods section.

## SUPPLEMENTAL MATERIAL

Table S1 spectrum.01344-26-s0001.pdf The clinical metadata for all 57 *S. maltophilia* isolates, listing each isolate (CASM ID) with its collection year (2018–2021), patient age (ranging from neonates noted in days and weeks up to 91 years), and sex. It is the patient-level context table for the cohort.

Table S2 spectrum.01344-26-s0002.pdf The Illumina short-read sequencing and assembly metrics for all 57 isolates, with one row per isolate giving the number of contigs, genome size in base pairs, mean short-read coverage, number of coding sequences, number of tRNAs, N50, GC content, genome completeness percentage, and the NCBI accession number.

Table S3 spectrum.01344-26-s0003.pdf The five isolates that also had Nanopore long-read sequencing (CASM7, CASM26, CASM33, CASM81, CASM106). It reports hybrid-assembly metrics (contigs, completeness, GC, genome size, N50) alongside the long-read-only completeness and mean coverage and points back to Table S2 for their short-read data.

ASM does not own the copyrights to Supplemental Material that may be linked to, or accessed through, an article. The authors have granted ASM a non-exclusive, world-wide license to publish the Supplemental Material files. Please contact the corresponding author directly for reuse.
