# Supplementary material for: Whole-genome characterization of clinical Stenotrophomonas maltophilia in Lebanon: sequence types, resistome, and T6SS diversity
Source: Microbiol Spectr. 2026 Sep 18;14(11):e01344-26. doi: 10.1128/spectrum.01344-26 (PMC13729162; doi:10.1128/spectrum.01344-26)
Supplement: Supplementary file 1 [file spectrum.01344-26-s0001.pdf]

Table S1. Metadata of the 57 clinical *Stenotrophomonas maltophilia* isolates, including collection year, patient age, and sex.

Sample#	Date	Age	Sex
CASM6	2018	76	F
CASM7	2018	27D	F
CASM8	2018	42	F
CASM10	2018	5W	F
CASM11	2018	78	F
CASM24	2018	67	M
CASM25	2018	86	M
CASM26	2018	88	M
CASM27	2019	17	F
CASM31	2019	57	M
CASM32	2019	33	F
CASM33	2019	20	F
CASM34	2019	33	F
CASM35	2019	66	M
CASM36	2019	7M	M

CASM37	2019	78	F
CASM43	2019	20	M
CASM44	2019	73	F
CASM48	2019	75	M
CASM51	2019	64	M
CASM52	2019	63	F
CASM54	2019	46	F
CASM55	2019	57	F
CASM56	2019	64	M
CASM68	2019	4	M
CASM70	2019	56	M
CASM72	2019	15	M
CASM76	2019	24	F
CASM78	2019	34	M
CASM79	2019	51	M
CASM80	2019	64	F
CASM81	2019	58	F

CASM82	2019	66	M
CASM85	2020	76	F
CASM87	2020	16	F
CASM88	2020	73	M
CASM89	2020	74	M
CASM90	2020	80	M
CASM91	2020	68	M
CASM92	2020	68	M
CASM94	2020	37	F
CASM95	2020	76	M
CASM97	2020	72	M
CASM98	2020	68	M
CASM99	2020	72	M
CASM100	2020	66	M
CASM101	2020	72	F
CASM102	2020	75	M
CASM106	2021	83	M

CASM107	2021	91	M
CASM109	2021	71	M
CASM110	2021	66	M
CASM111	2021	66	F
CASM112	2021	71	M
CASM113	2021	60	M
CASM114	2021	71	M
CASM115	2021	55	M

D:days, W:weeks; F:female, M:male
