# Supplementary material for: Whole-genome characterization of clinical Stenotrophomonas maltophilia in Lebanon: sequence types, resistome, and T6SS diversity
Source: Microbiol Spectr. 2026 Sep 18;14(11):e01344-26. doi: 10.1128/spectrum.01344-26 (PMC13729162; doi:10.1128/spectrum.01344-26)
Supplement: Supplementary file 2 [file spectrum.01344-26-s0002.pdf]

**Table S2. Illumina Sequencing data, Short reads assembly metrics and NCBI accession numbers for all isolates.**
Number of contigs, genome size (bp), mean coverage, number of coding sequence, number of tRNA, N50(bp),GC content (%), genome completeness (%), and accession numbers are shown for each of the 57 *S. maltophilia* isolates.

| Sample ID# | # of contigs | Genome Size (bp) | Mean Coverage | # of Coding Sequence | # of tRNA | N50(bp) | GC% | Completeness % | Accession number |
|---|---|---|---|---|---|---|---|---|---|
| CASM6 | 105 | 5110577 | 40.3 | 4604 | 75 | 101222 | 66.11 | 99.1% | JAVTIG000000000 |
| CASM7 | 84 | 4586931 | 101.1 | 4124 | 73 | 258200 | 66.5 | 99.6% | JBMRFG000000000 |
| CASM8 | 75 | 3936337 | 259.0 | 3500 | 77 | 132500 | 67 | 99.3% | JBMQIY000000000 |
| CASM10 | 283 | 4560588 | 12.6 | 4130 | 73 | 29200 | 66.5 | 99.5% | JBMREU000000000 |
| CASM11 | 113 | 4512636 | 49.6 | 4019 | 81 | 120008 | 66.5 | 99.2% | JBMREV000000000 |
| CASM24 | 1625 | 4209386 | 2.1 | 3942 | 64 | 4226 | 66.5 | 99.2% | JBMRFH000000000 |
| CASM25 | 49 | 4516545 | 118.6 | 4047 | 76 | 307430 | 66.5 | 99.3% | JBMRFU000000000 |
| CASM26 | 113 | 4815084 | 66.1 | 4369 | 74 | 211108 | 66 | 99.3% | JBMRFV000000000 |
| CASM27 | 104 | 4810090 | 151.2 | 4373 | 74 | 149913 | 66 | 99.3% | JBMQIV000000000 |
| CASM31 | 97 | 4487575 | 50.1 | 4046 | 75 | 116836 | 66.5 | 99.3% | JBMRFW000000000 |
| CASM32 | 78 | 4767292 | 41.3 | 4292 | 74 | 134097 | 66.5 | 99.1% | JBMREW000000000 |
| CASM33 | 48 | 4771656 | 99.1 | 4294 | 74 | 474931 | 66.3 | 99.1% | JAVTOB000000000 |
| CASM34 | 46 | 4767295 | 77.4 | 4295 | 74 | 240758 | 66.3 | 99.2% | JAVTOC000000000 |
| CASM35 | 72 | 4774792 | 44.0 | 4302 | 74 | 190963 | 66.3 | 99.2% | JBMRFX000000000 |
| CASM36 | 123 | 4742202 | 61.4 | 4308 | 78 | 123658 | 66.5 | 99.2% | JBMREX000000000 |
| CASM37 | 121 | 4739422 | 44.5 | 4316 | 78 | 99395 | 66.4 | 99.2% | JBMRFY000000000 |

| | | | | | | | | | |
|---|---|---|---|---|---|---|---|---|---|
| CASM43 | 77 | 4417787 | 96.3 | 3947 | 73 | 128908 | 66.76 | 99.3% | JAVTOF000000000 |
| CASM44 | 53 | 4672770 | 48.0 | 4236 | 74 | 319331 | 66.5 | 99.2% | JBMREY000000000 |
| CASM48 | 74 | 4848976 | 27.8 | 4388 | 77 | 129738 | 66.5 | 99.2% | JBMRFI000000000 |
| CASM51 | 96 | 4713492 | 52.2 | 4210 | 79 | 96501 | 66.5 | 99.3% | JBMREZ000000000 |
| CASM52 | 78 | 4754472 | 106.9 | 4320 | 74 | 226471 | 66.77 | 99.1% | JAVTOG000000000 |
| CASM54 | 839 | 4637994 | 298.0 | 4417 | 62 | 9810 | 66.71 | 87.4% | JBMQIZ000000000 |
| CASM55 | 70 | 4473813 | 46.9 | 4050 | 74 | 243992 | 66.5 | 99.2% | JBMRFA000000000 |
| CASM56 | 63 | 4473666 | 60.6 | 4045 | 74 | 245906 | 66.5 | 99.3% | JBMRFZ000000000 |
| CASM68 | 83 | 4398727 | 40.9 | 3901 | 73 | 119765 | 67 | 99% | JBMRFB000000000 |
| CASM70 | 62 | 4288450 | 137.2 | 3772 | 70 | 365020 | 67 | 99.3% | JBMRGA000000000 |
| CASM72 | 70 | 4290417 | 87.2 | 3774 | 70 | 283031 | 67 | 99.3% | JBMRGB000000000 |
| CASM76 | 57 | 4648336 | 118.5 | 4148 | 73 | 173894 | 66.71 | 99.2% | JAVTOH000000000 |
| CASM78 | 83 | 4344201 | 41.4 | 3871 | 74 | 128874 | 67 | 99.2% | JBMRFC000000000 |
| CASM79 | 94 | 4451493 | 71.9 | 3990 | 74 | 153058 | 67 | 99.1% | JBMRGC000000000 |
| CASM80 | 117 | 4612823 | 42.9 | 4134 | 76 | 86312 | 66.5 | 99.2% | JBMRGD000000000 |
| CASM81 | 75 | 4624749 | 93.1 | 4203 | 74 | 123217 | 66.61 | 99.3% | JAVTOI000000000 |
| CASM82 | 88 | 4761113 | 24.9 | 4281 | 77 | 117448 | 66.5 | 99.5% | JBMRFJ000000000 |
| CASM85 | 49 | 4768530 | 43.1 | 4240 | 73 | 254187 | 66.49 | 99% | JAVTOJ000000000 |
| CASM87 | 108 | 4571293 | 166.8 | 4070 | 78 | 110576 | 66.48 | 99.1% | JAVTOK000000000 |
| CASM88 | 104 | 4545586 | 37.5 | 4055 | 74 | 83101 | 66.6 | 99% | JBMRFM000000000 |

| | | | | | | | | | |
|---|---|---|---|---|---|---|---|---|---|
| CASM89 | 89 | 4875870 | 180.3 | 4465 | 80 | 244517 | 66 | 99.5% | JBMRFN000000000 |
| CASM90 | 52 | 4428229 | 169.5 | 3952 | 78 | 256513 | 66.5 | 99.2% | JBMRFO000000000 |
| CASM91 | 47 | 4477773 | 97.8 | 3980 | 71 | 275563 | 66.5 | 99.2% | JBMRGE000000000 |
| CASM92 | 50 | 4479211 | 72.0 | 3984 | 73 | 248059 | 66.5 | 99.1% | JBMRGF000000000 |
| CASM94 | 94 | 4514521 | 56.6 | 4051 | 77 | 119301 | 66.5 | 99.5% | JBMRGG000000000 |
| CASM95 | 61 | 4507977 | 131.1 | 4048 | 77 | 191879 | 66.5 | 99.3% | JBMRFP000000000 |
| CASM97 | 57 | 4757785 | 62.5 | 4230 | 73 | 240194 | 66.5 | 99.2% | JBMRFK000000000 |
| CASM98 | 93 | 4982182 | 260.7 | 4498 | 72 | 146446 | 65.5 | 99.2% | JBMRFQ000000000 |
| CASM99 | 95 | 4502021 | 72.5 | 4011 | 76 | 139835 | 66.5 | 99.2% | JBMRFD000000000 |
| CASM100 | 62 | 4573660 | 42.6 | 4129 | 75 | 226561 | 66.5 | 99.5% | JBMRFL000000000 |
| CASM101 | 53 | 4573726 | 107.4 | 4127 | 76 | 236388 | 66.5 | 99.5% | JBMRFR000000000 |
| CASM102 | 52 | 4398915 | 402.4 | 3928 | 73 | 356900 | 66.5 | 99.2% | JBMQIX000000000 |
| CASM106 | 109 | 4818912 | 63.3 | 4364 | 75 | 540736 | 66.5 | 99.2% | JBMRFS000000000 |
| CASM107 | 46 | 4475708 | 78.3 | 3984 | 71 | 298104 | 66.5 | 99.1% | JBMRGH000000000 |
| CASM109 | 38 | 4436915 | 145.9 | 3969 | 73 | 281277 | 67 | 99.2% | JBMQIW000000000 |
| CASM110 | 68 | 4542899 | 34.1 | 4128 | 71 | 164326 | 67 | 99.1% | JBMRGI000000000 |
| CASM111 | 103 | 4916928 | 171.6 | 4465 | 83 | 207771 | 66 | 99% | JBMRFT000000000 |
| CASM112 | 68 | 4437547 | 33.2 | 3968 | 75 | 193351 | 67 | 99.5% | JBMRFE000000000 |
| CASM113 | 111 | 5008973 | 29.3 | 4560 | 72 | 100417 | 66 | 99.3% | JBMRFF000000000 |
| CASM114 | 65 | 4442247 | 53.7 | 3972 | 73 | 161299 | 67 | 99.1% | JBMRGJ000000000 |

| | | | | | | | | | |
|---|---|---|---|---|---|---|---|---|---|
| CASM115 | 3686 | 5637467 | 52.2 | 4507 | 65 | 2325 | 66.5 | 99.2% | JBWGIN000000000 |
