# Supplementary material for: Whole-genome characterization of clinical Stenotrophomonas maltophilia in Lebanon: sequence types, resistome, and T6SS diversity
Source: Microbiol Spectr. 2026 Sep 18;14(11):e01344-26. doi: 10.1128/spectrum.01344-26 (PMC13729162; doi:10.1128/spectrum.01344-26)
Supplement: Supplementary file 3 [file spectrum.01344-26-s0003.pdf]

Table S3 Nanopore sequencing and hybrid assembly quality metrics for the five selected isolates.
Assembly quality metrics include the number of contigs, genome size, N50, GC content, and genome completeness for the hybrid assemblies. Genome completeness and mean coverage are also reported for the corresponding Nanopore only assemblies (long reads). (short reads data for these 5 isolates are in table S2)

	Hybrid Assembly Data					Long Assembly Data	
**Sample ID#**	**Contigs#**	**Completeness %**	**GC%**	**Genome Size (bp)**	**N50 value**	**Completeness %**	**Mean Coverage**
**CASM7**	65	99.5	66.36	4572407	186710	49.6	91
**CASM26**	121	98.4	66.16	4760117	86292	59.5	59
**CASM33**	82	98.5	66.3	4764272	106333	36.1	17
**CASM81**	35	97.6	66.66	4783707	193444	89.7	84
**CASM106**	88	98.8	66.7	4758901	121466	45.2	17
